# Supplementary material for: The force-length relation of the young adult human tibialis anterior
Source: PeerJ. 2023 Jul 13;11:e15693. doi: 10.7717/peerj.15693 (PMC10350298; doi:10.7717/peerj.15693)
Supplement: Supplemental Information 1 [file peerj-11-15693-s001.docx]

**Supplemental Article S1.**

**Dorsiflexion attachment.**

The custom-made dorsiflexion attachment (see photo below) consisted of a U-shaped frame made of two vertical metal rods (5 mm diameter and 100 mm length), one horizontal metal rod (8 mm diameter and 130 mm length), and four 3D printed plastic blocks (30 mm length, 30 mm width, and 30 or 65 mm height; printed with 1.5 mm diameter polylactic acid filament, CREALITY, Shenzhen, China) that allowed the frame to be attached to the dynamometer’s dorsi/plantar flexion adapter and tightly secured over the metatarsals. The horizontal rod above the metatarsals was surrounded by a 3D printed hollow plastic cylinder (8 mm inner diameter and 20 mm outer diameter) and a hollow foam cylinder (20 mm inner diameter and 38 mm outer diameter) to minimize participant discomfort during voluntary dorsiflexion contractions.


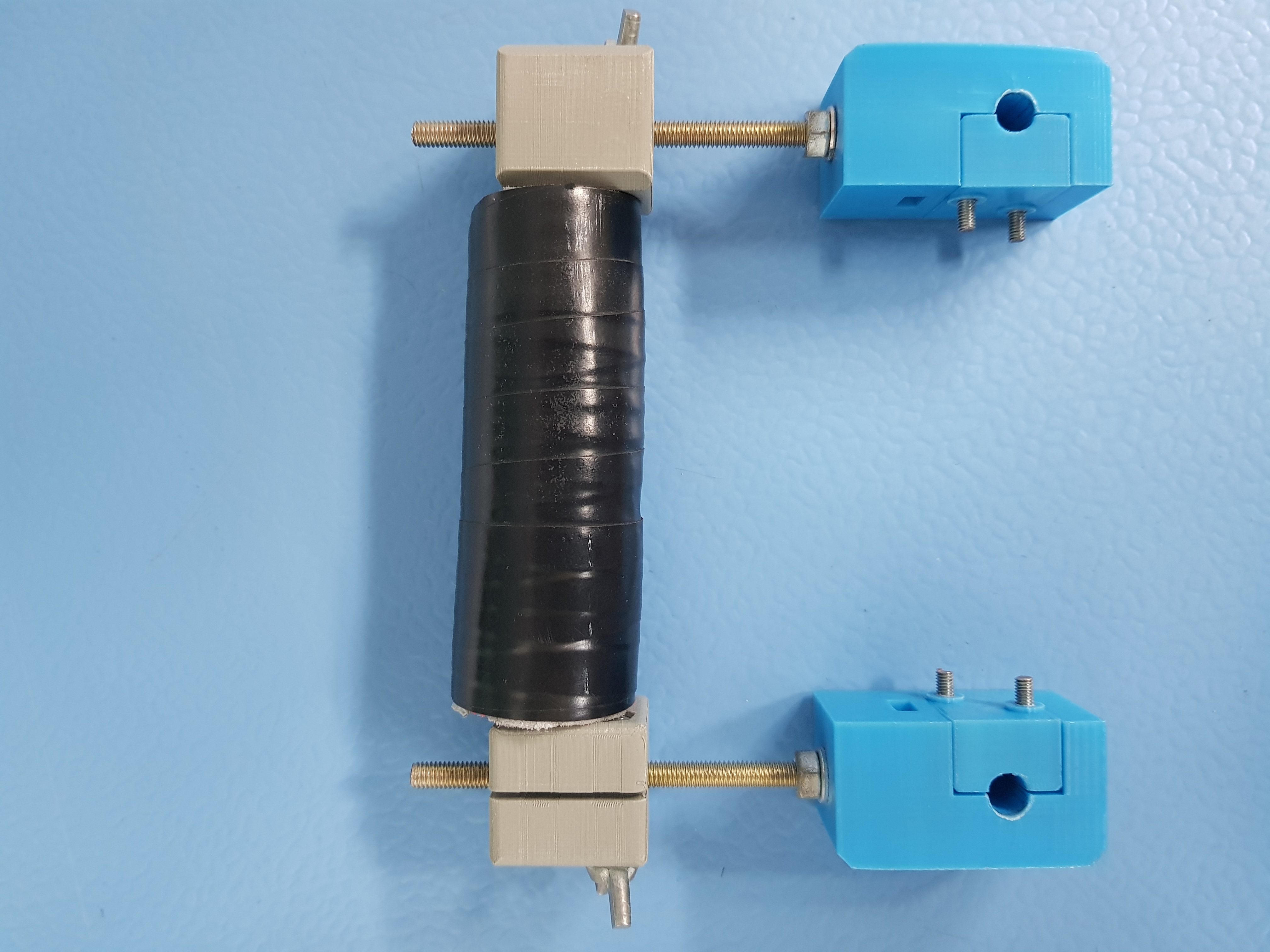


**Photo of the dorsiflexion attachment.**
