# Supplementary material for: The force-length relation of the young adult human tibialis anterior
Source: PeerJ. 2023 Jul 13;11:e15693. doi: 10.7717/peerj.15693 (PMC10350298; doi:10.7717/peerj.15693)
Supplement: Supplemental Information 2 [file peerj-11-15693-s002.docx]

**Supplemental Article S2.**

**Brightness-mode ultrasound and bipolar surface electromyography techniques.**

Tibialis anterior (TA) muscle fascicles were imaged using a linear-array, flat-shaped, 128‐element transducer that was operating at 6.5 MHz and coupled to a PC‐based ultrasound system (ArtUs EXT-1H, TELEMED, Vilnius, Lithuania). Ultrasound transmission gel (Aquasonic 100, Parker Laboratories Inc., Fairfield, New Jersey, United States) was applied to the skin and transducer face, and to obtain clearer images of TA’s muscle fascicles and its aponeuroses, a large latex glove (Unigloves, Troisdorf, Germany) was half-filled with the same gel and positioned between the skin and transducer face. Once clear images with no shadows were obtained at rest and during contraction of TA’s muscle fascicles and its three aponeuroses, which were visible as oblique white line-like structures and thicker horizontal white line-like structures, respectively, the transducer was secured distal to the most proximal end of TA’s central aponeurosis using self-adhesive bandage. This arrangement limited transducer movement relative to the skin. Ultrasound images were captured at either ~34 fps (five participants with a high line density) or ~137 fps (eleven participants) with a field of view (width × height) of 60 mm x 50 mm and a resolution of 0.065 mm per pixel using Echo Wave II software (4.1.1 64-bit version, TELEMED). The frame rate was lower for five participants as towards the end of testing it was observed that the tracking algorithm (Supplemental Article S4) underestimated fascicle length changes less when the frame rate was ~30 fps. One fixed focal zone around the central aponeurosis was used, and image enhancement (method 4) and speckle reduction (level 3 PureView) were enabled to improve the fascicle visibility. The ultrasound system generated a digital pulse via its ultrasound frame output once each ultrasound image was collected, and this signal was recorded at 2 kHz using a 16-bit analog-to-digital converter (Power1401-3) and Spike2 (8.23 64-bit version) data collection system (Cambridge Electronic Design Ltd., Cambridge, United Kingdom), which had a ±5 V input range.

The myoelectric signal from the right TA was recorded with a NeuroLog system (NL905, Digitimer Ltd., Welwyn Garden City, United Kingdom) using two electrodes with a 2 cm inter-electrode distance over the distal aspect of the TA's superficial muscle compartment. Brightness-mode ultrasound was used to ensure that the electrodes were aligned with the direction of muscle fascicles and away from the muscle’s borders. A single reference electrode was taped (1.25 cm width surgical tape, 3M Transpore, St. Paul, Minnesota, United States) to the skin over the right fibular head. Electrode placement was preceded by standard skin preparation that involved shaving (Teqler, Wecker, Luxembourg), abrading (Nuprep, Weaver and Company, Aurora, United States) and cleaning (Sterillium, HARTMANN, Heidenheim, Germany). The myoelectric signal was amplified 2000 times and band‐pass analog filtered from 10-500 Hz, prior to being sampled at 2 kHz using the same Spike2 data collection system described above. The EMG signal was smoothed using a centred moving root-mean-square (RMS) amplitude calculation of 125 ms after the DC offset was removed (rmsDC.m; doi:10.5281/zenodo.7411400).
