# Supplementary material for: The force-length relation of the young adult human tibialis anterior
Source: PeerJ. 2023 Jul 13;11:e15693. doi: 10.7717/peerj.15693 (PMC10350298; doi:10.7717/peerj.15693)
Supplement: Supplemental Information 3 [file peerj-11-15693-s003.docx]

**Supplemental Article S3.**

**Data export.**

A custom-written script (spike2mat.s2s; doi:10.5281/zenodo.7411280) in Spike2 (8.23 64-bit version, Cambridge Electronic Design Ltd., Cambridge, United Kingdom) was used to export individual trials from a recording of the entire experimental session as separate files with a .mat extension. The recorded digital signals from each trial were cropped between a common start and end time using the timestamps of the first and last digital pulses from each ultrasound recording. The ultrasound videos, which were stored as files with a .tvd extension, were cropped to only include the field of view of the ultrasound images and converted to files with .mp4 and .mat extensions using a custom-written script (convertTVD2ALL.m; doi:10.5281/zenodo.7411280) in MATLAB (R2022a 64-bit version, Mathworks, Natick, Massachusetts, United States). The files with a .mat extension contained information about the video cropping, the frame rate and number of frames, the timestamps, and the distance per pixel. To confirm that each exported trial from Spike2 had a corresponding cropped video, the durations all files were calculated and compared. Data was considered synchronized and subsequently combined when the duration difference between files was no more than one frame (<0.03 s).
