# Supplementary material for: The force-length relation of the young adult human tibialis anterior
Source: PeerJ. 2023 Jul 13;11:e15693. doi: 10.7717/peerj.15693 (PMC10350298; doi:10.7717/peerj.15693)
Supplement: Supplemental Information 4 [file peerj-11-15693-s004.docx]

**Supplemental Article S4.**

**Ultrasound tracking.**

An updated version (UltraTrack_v5_3.m; doi:10.5281/zenodo.7411280) of UltraTrack (Farris and Lichtwark, 2016) was used to estimate fascicle length changes from B-mode ultrasound recordings. Briefly, the updated version differs from the previous version because it tracks detected feature points across sequential images using a Kanade-Lucas-Tomasi (KLT) feature-point tracking algorithm (Lucas and Kanade, 1981; Shi and Tomasi, 1994). The KLT algorithm was implemented instead of the Lucas-Kanade algorithm (Lucas and Kanade, 1981) because it results in lower root-mean-squared errors between automatically-determined and manually-defined fascicle lengths compared with the earlier version of UltraTrack as mentioned in Drazan et al. (2019).

Absolute fascicle length was first calculated automatically from one ultrasound image captured before the start of the sixth passive ankle plantar flexion rotation trial using open-source MATLAB code (https://github.com/JasperVerheul/hybrid-muscle-tracking). The superficial and central or central and deep aponeuroses were initially identified by the user and the image was cropped to 1 mm around each aponeurosis. Similar to previous work (van der Zee and Kuo, 2022), vessel-like structures within the cropped images were then determined using a Hessian-based Frangi vesselness filter (Frangi et al., 1998) that has been implemented in MATLAB (https://www.mathworks.com/matlabcentral/fileexchange/24409-hessian-based-frangi-vesselness-filter). A Frangi scale range of 1-2 mm was used and the cropped and filtered images were binarized using adaptive thresholding. The centroid and orientation of the vessel structures with the largest perimeter in each image were then used to define a line representing each aponeurosis.

Using the aponeurosis lines, a region of interest was defined over the entire width of the image as the area 1 mm below and above the upper (i.e. superficial or central) and lower (i.e. central or deep) aponeuroses, respectively. The open-source code was modified to implement a Jerman enhancement filter (https://www.mathworks.com/matlabcentral/fileexchange/63171-jerman-enhancement-filter) to improve the filter response for vessels of varying contrasts and sizes (Jerman et al., 2016a, 2016b). The region of interest was then stretched vertically by a factor of three using bicubic interpolation and the Hough transform (Hough, 1962) was applied. Hough lines with orientations of 45° and less than 0-25° were subsequently discarded. Note that a range is provided as the exact value was image dependent, and the lower limit was changed when the dominant fascicle line obviously disagreed with what was visually expected. The default parameters of the open-source code were then used to determine a dominant fascicle orientation from that image and region of interest. Linear extrapolation was performed to find the fascicle endpoints, which reflected the intersections between the previously determined aponeurosis lines and the dominant fascicle line, which was centred within the region of interest.

Fascicle length changes were subsequently estimated by tracking the automatically-defined fascicle endpoints and region of interest locations forward one image at a time using a KLT feature-point tracking algorithm (Lucas and Kanade, 1981; Shi and Tomasi, 1994). This algorithm tracked feature points from one image to the next using a point-tracker object that performed up to 50 iterations, had four pyramid levels, and a block size (width x height) of 21 x 71 pixels. Feature points were renewed for each image and were detected as corners within each image from a moving region of interest using a minimum eigenvalue criterion (Shi and Tomasi, 1994). Matched feature-point pairs between successive images were then used to estimate a two-dimensional affine geometric transformation, which required a desired confidence of 99% for finding the maximum number of matched pairs, and allowed a maximum distance of 50 pixels from one point to the projected location of its corresponding point.

Absolute fascicle lengths from other trials of the same participant were calculated as follows. The mean filtered angle over the first second of each trial was calculated, and the closest filtered angle during the previously tracked 5°·s^-1^ passive rotation trial was determined. The fascicle endpoints and region of interest locations at that angle were then saved and loaded into the trial of interest. That trial was subsequently tracked forward with a moving region of interest to estimate fascicle length changes. Absolute fascicle lengths and fascicle angles (relative to the horizontal) from each tracked image were saved and synchronized with the recorded digital signals of the corresponding trial for later analysis.

**References**

Drazan, J.F., Hullfish, T.J., Baxter, J.R., 2019. An automatic fascicle tracking algorithm quantifying gastrocnemius architecture during maximal effort contractions. PeerJ 7, e7120. https://doi.org/10.7717/peerj.7120

Farris, D.J., Lichtwark, G.A., 2016. UltraTrack: Software for semi-automated tracking of muscle fascicles in sequences of B-mode ultrasound images. Computer Methods and Programs in Biomedicine 128, 111–118. https://doi.org/10.1016/j.cmpb.2016.02.016

Frangi, A.F., Niessen, W.J., Vincken, K.L., Viergever, M.A., 1998. Multiscale vessel enhancement filtering, in: Wells, W.M., Colchester, A., Delp, S. (Eds.), Medical Image Computing and Computer-Assisted Intervention — MICCAI’98, Lecture Notes in Computer Science. Springer Berlin Heidelberg, Berlin, Heidelberg, pp. 130–137. https://doi.org/10.1007/BFb0056195

Hough, P.V.C., 1962. Method and means for recognizing complex patterns. US3069654A.

Jerman, T., Pernus, F., Likar, B., Spiclin, Z., 2016a. Enhancement of vascular structures in 3D and 2D angiographic images. IEEE Trans. Med. Imaging 35, 2107–2118. https://doi.org/10.1109/TMI.2016.2550102

Jerman, T., Pernus, F., Likar, B., Spiclin, Z., 2016b. Blob enhancement and visualization for improved intracranial aneurysm detection. IEEE Trans. Visual. Comput. Graphics 22, 1705–1717. https://doi.org/10.1109/TVCG.2015.2446493

Lucas, B., Kanade, T., 1981. An iterative image registration technique with an application to stereo vision, in: [No Source Information Available]. Presented at the Proceedings of the 7th International Joint Conference on Artificial Intelligence, pp. 121–130.

Shi, J., Tomasi, C., 1994. Good features to track, in: Proceedings of IEEE Conference on Computer Vision and Pattern Recognition CVPR-94. Presented at the 1994 Proceedings of IEEE Conference on Computer Vision and Pattern Recognition, IEEE Comput. Soc. Press, Seattle, WA, USA, pp. 593–600. https://doi.org/10.1109/CVPR.1994.323794

van der Zee, T.J., Kuo, A.D., 2022. TimTrack: A drift-free algorithm for estimating geometric muscle features from ultrasound images. PLoS ONE 17, e0265752. https://doi.org/10.1371/journal.pone.0265752
