# Supplementary material for: The force-length relation of the young adult human tibialis anterior
Source: PeerJ. 2023 Jul 13;11:e15693. doi: 10.7717/peerj.15693 (PMC10350298; doi:10.7717/peerj.15693)
Supplement: Supplemental Information 5 [file peerj-11-15693-s005.docx]

**Supplemental Article S5.**

**Active torque calculation.**

To calculate active net ankle joint torque, a steady-state passive joint torque-joint angle fit was first constructed, which involved fitting a third-order polynomial to the mean ankle joint angle and net ankle joint torque (filtered) data calculated over one second at the start of each trial when participants were instructed to relax. If the EMG root-mean-squared amplitude during the 1-s window exceeded the minimum amplitude from the entire trial by a factor of two, the trial was excluded from the fit. Additionally, third-order polynomials were fit to the (filtered) angle and torque data from the sixth passive ankle dorsiflexion and plantar flexion rotations. The three fits were then evaluated over the ankle joint angles achieved during the passive rotations, and the torque values calculated from the steady-state fit were confirmed to fall between the torque values calculated from the passive rotation fits (passive_torque.m; doi:10.5281/zenodo.7411400). Active net ankle joint torque from each trial was then calculated by evaluating the steady-state fit at the angles recorded during the trial and then subtracting the calculated passive net ankle joint torque values from the recorded net ankle joint torque (active_torque_mvc.m; doi:10.5281/zenodo.7411400).
