# Supplementary figures and images for: The force-length relation of the young adult human tibialis anterior
Source: PeerJ. 2023 Jul 13;11:e15693. doi: 10.7717/peerj.15693 (PMC10350298; doi:10.7717/peerj.15693)

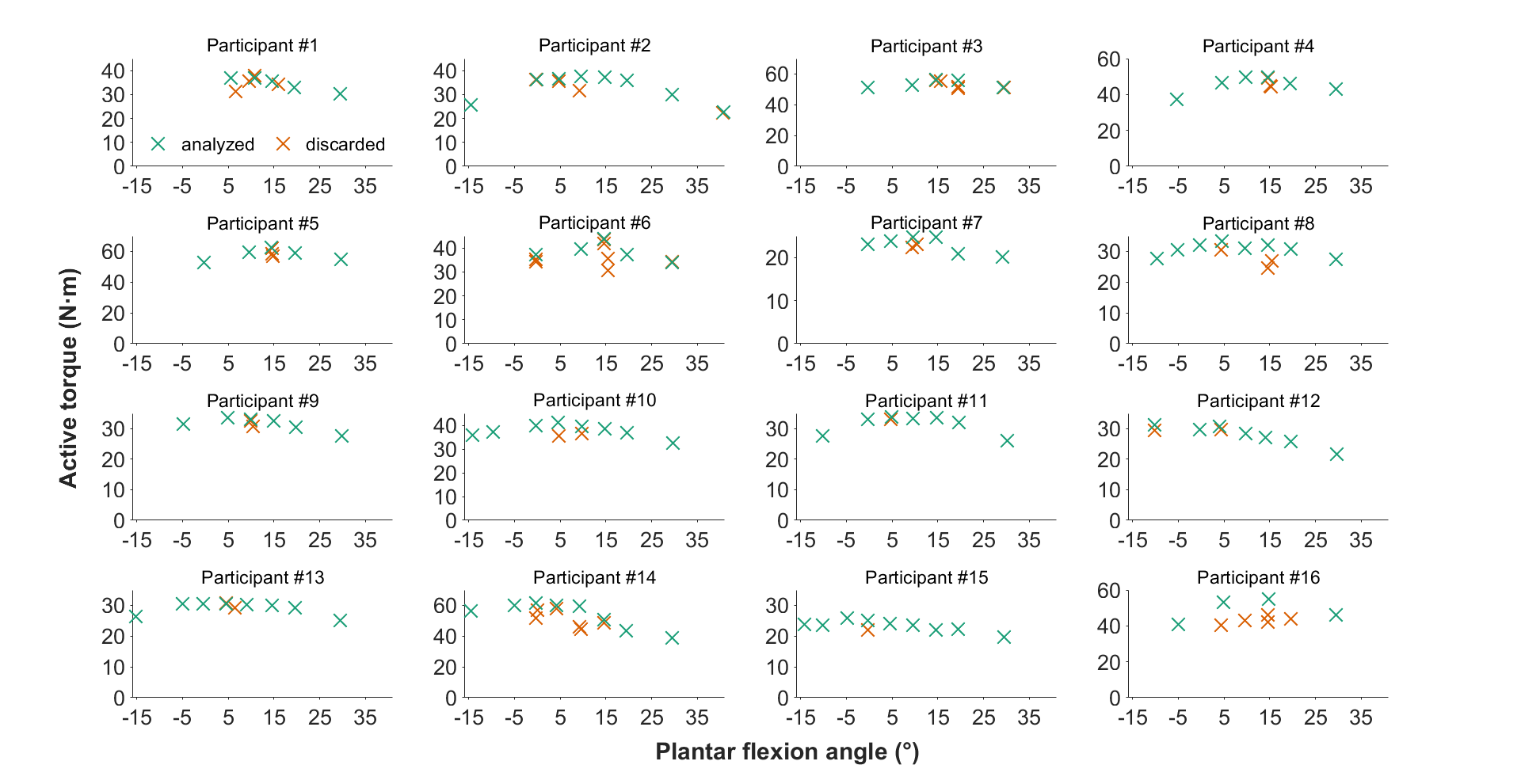

Supplement: Figure S1 — Note that because participants #1, #4, #9, and #16 did not perform maximal voluntary contractions at 0° plantar flexion, their datasets were excluded from the analysis. The maximal voluntary contractions at −15° (n = 5), −10° (n = 5), −5° (n = 7), and 40° (n = 1) plantar flexion were also excluded from the analysis. [file peerj-11-15693-s006.png]
